# Resistive Switching Characteristic Improvement in a Single-Walled Carbon Nanotube Random Network Embedded Hydrogen Silsesquioxane Thin Films for Flexible Memristors

**DOI:** 10.3390/ijms22073390

**Published:** 2021-03-25

**Authors:** Shin-Yi Min, Won-Ju Cho

**Affiliations:** Department of Electronic Materials Engineering, Kwangwoon University, Gwangun-ro 20, Nowon-gu, Seoul 01897, Korea; kkuregi1234@naver.com

**Keywords:** hydrogen silsesquioxane, single-walled carbon nanotube random network, nonvolatile multiple-resistance states, memristor, synaptic weight modulation

## Abstract

In this study, we evaluated the improved memristive switching characteristics of hydrogen silsesquioxane (HSQ) nanocomposites embedded with a single-walled carbon nanotube (SWCNT) random network. A low-temperature solution process was implemented using a flexible memristor device on a polyethylene naphthalate (PEN) substrate. The difference in the resistive switching (RS) behavior due to the presence of the SWCNT random network was analyzed by the current transport mechanism. Such a random network not only improves the RS operation but also facilitates a stable multilevel RS performance. The multiple-resistance states exhibited highly reliable nonvolatile retention properties over 10^4^ s at room temperature (25 °C) and at a high temperature (85 °C), showing the possibility of an analog synaptic weight modulation. Consequently, the gradual weight potentiation/depression was realized through 3 × 10^2^ synaptic stimulation pulses. These findings suggest that the embedded SWCNT random network can improve the synaptic weight modulation characteristics with high stability for an artificial synapse and hence can be used in future neuromorphic circuits.

## 1. Introduction

Over the past few years, the fields of information science and electronics technology have achieved remarkable advances to overcome the conventional Von Neumann architecture [1,2]. Particularly, high computing efficiency is regarded as a new benchmark in processing big data collected from various information platforms, such as the Internet of things, autonomous vehicles, and smart sensors [3,4]. Because memory units are the most essential building blocks for electronic devices, various types of next-generation memory devices have been developed to overcome physical limitations and low storage capacity [5,6]. Among them, memristor-based memory elements have attracted considerable attention. Two-terminal memristor devices with a metal–insulator–metal (MIM) structure offer potential advantages in terms of geometrical simplicity, nonvolatile data storage, and low operating power consumption [7,8]. They can modulate the conductance state to facilitate multi-bit working memory and enable analog neuromorphic computing for emerging electronic applications [9,10,11,12]. To modulate the conductance of the insulating layer in the memristor of the MIM structure, various materials based on phase change or conductive filaments for the resistive switching (RS) layer have been proposed [5,13]. The oxide-based conductive filament RS layer exhibits excellent RS properties, good thermal stability, and stable mechanical strength compared to other materials [14,15,16,17,18]. Meanwhile, the RS properties of silicon-oxide-based materials have been reported since the 1960s through various configurations and structures, such as amorphous silicon oxide, silicon-rich silica (SiO_x_) nanopillars, and metal-dispersed SiO_2_ films [19,20,21,22,23,24]. In particular, memristor devices using SiO_x_-based spin-on-glass (SOG) silsesquioxane as the RS layer have been recently reported [25,26,27,28].

In this study, we propose a flexible memristor device with excellent RS characteristics fabricated on a polyethylene naphthalate (PEN) substrate using a thin film of hydrogen silsesquioxane (HSQ) embedded with a single-walled carbon nanotube (SWCNT) random network as the RS layer. HSQ is a siloxane-based inorganic polymer SOG material with a chemical composition of (HSiO_3/2_)*_n_* (*n* = 2, 3, 4, …), whose abundant oxygen atoms lead to oxygen vacancy-rich conductive filaments and induce RS behavior [29,30]. In addition, HSQ is easily spin-coated on the surface of a semiconductor or metal through a low-temperature solution process and is widely used in interlayer dielectrics due to its low dielectric constant, atmospheric stability, thickness uniformity, excellent planarization, and gap-fill capabilities [31]. Meanwhile, an SWCNT random network can be simply formed through a solution drop-casting method and has excellent flexibility, transparency, mechanical strength, and electrical properties [32,33,34,35]. This finding suggests that an SWCNT random network embedded in HSQ thin films enables the fabrication of flexible memristor devices with excellent RS characteristics. Accordingly, we systematically investigated the effects of an SWCNT random network embedded in HSQ memristor devices on the RS operations and synaptic behaviors.

## 2. Experimental

### 2.1. Materials

The materials used in this study are as follows: PEN substrate (125 μm thick; AMG Co., Seoul, Korea), glass substrate (7059 glass; Corning Inc., New York, USA), cool-off-type adhesive (Intelimer^TM^ Tape CS2325NA4; Nitta Corp., Tokyo, Japan), Ti pellet (purity > 99.999%; TIFINE Co., Incheon, Korea), Pt pellet (purity > 99.95%; TIFINE Co., Incheon, Korea), poly-L-lysine solution (0.1% (*w*/*v*) in H_2_O; Sigma Aldrich, Saint Louis, USA), SWCNT solution (IsoNanotubeS; average diameter = 1.4 nm, average length = 300 nm; Nanointegris, Quebec, Canada), and HSQ solution (Fox-12; Dow Corning Corp., Michigan, USA).

### 2.2. Flexible Memristor Device Fabrication

Prior to fabricating the flexible memristor devices, the PEN substrate was attached to a rigid glass substrate using a cool-off-type adhesive to avoid shrinkage and thermal expansion problems during the process. The flexible PEN substrate was easily separated from the glass substrate without physical destruction at low temperatures (<5 °C) after the final fabrication process. To form the bottom electrode (BE) layer of the MIM structure on the PEN substrate, a 100-nm-thick indium tin oxide (ITO) film was deposited using a radio-frequency (RF) magnetron sputtering system. The sputtering process was carried out at room temperature with Ar/O_2_ mixed gas (Ar:O_2_ = 20:1 sccm). 3.0 mTorr working pressure, and 100 W RF power. Subsequently, deposition of 10-nm-thick Ti and 10-nm-thick Pt thin films followed by using an electron-beam (E-beam) evaporator. The embedment of the SWCNT random network in HSQ thin-film nanocomposites, which is the most crucial part for the RS operation in memristor devices, was performed as follows: First, a 20-nm-thick SiO_2_ thin film was deposited on the BE via RF magnetron sputtering. The sputtering process was conducted at room temperature with an Ar/O_2_ mixed gas (Ar:O_2_ = 30:2 sccm), 4.2 mTorr working pressure, and 100 W RF power. This SiO_2_ thin film can be easily functionalized in the amine-terminated layer using a poly-L-lysine solution and can provide an effective adhesive layer to the SWCNT random network [21,36]. The poly-L-lysine solution was drop-casted on SiO_2_ thin film and was kept for 30 min at room temperature. Then, the samples were washed in deionized (DI) water and dried by N_2_ gas blowing. Second, the SWCNT random network was developed by drop-casting an SWCNT solution on the amine-terminated SiO_2_ layer. The SWCNT solution drop-casted samples were kept for 1 h at room temperature to functionalize the SWCNT random network with amine groups. Then, the samples were also washed in DI water and dried by N_2_ gas blowing. Finally, The HSQ solution was then spin-coated at 6000 rpm for 30 s, and the baking process was then performed with a convection oven system for 10 min at 120 °C, which is a non-destructive condition for the PEN substrate. As a result, the RS layer of a 100-nm-thick SWCNT random network embedded HSQ thin-film nanocomposites was uniformly formed through the high gap-fill capability of HSQ. Finally, to form the top electrode (TE) of the MIM structure, a 200-μm-diameter and 100-nm-thick Ti film was deposited on the RS layer using an E-beam evaporator and a shadow mask. Furthermore, to investigate the influence of the SWCNT random network on the memristive switching characteristics, memristors without SWCNTs were also prepared.

**Figure 1:**
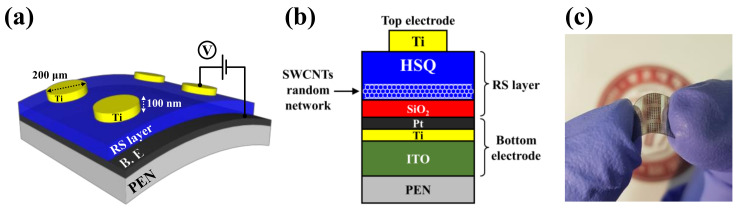
Hydrogen silsesquioxane (HSQ) memristor with an embedded single-walled carbon nanotube (SWCNT) random network fabricated on a flexible polyethylene naphthalate (PEN) substrate: (**a**) schematic diagram, (**b**) cross-sectional detail, and (**c**) photographic image.

Figure 1a–c shows the schematic, cross-sectional detail, and photographic images of an HSQ memristor device with an embedded SWCNT random network fabricated on a flexible PEN substrate, respectively.

**Figure 2:**
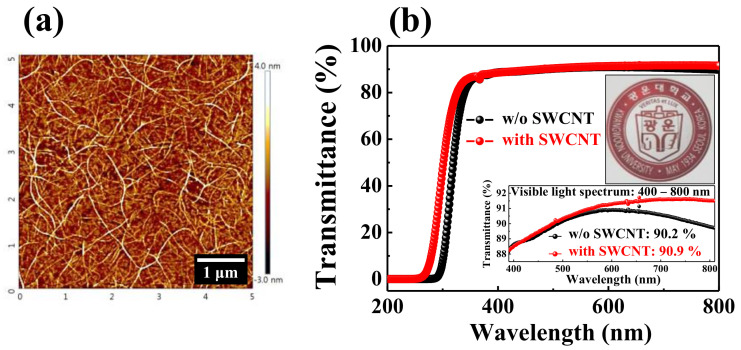
(**a**) Topographic image of the SWCNT random network observed with an atomic force microscope (AFM) (5 × 5 μm^2^). (**b**) Optical transmittance spectra of the resistive switching (RS) layer w/o and w/ the embedded SWCNT random network on the glass substrate. The insets depict the optical transmittance in the visible-light wavelength spectrum (400–800 nm) and photographic image of the RS layer w/ the embedded SWCNT random network.

The topographic image of the SWCNT random network observed with an atomic force microscope (AFM) (5 × 5 μm^2^) is shown in Figure 2a. The AFM observation demonstrated that the SWCNT random network was successfully developed on the amine-terminated thin SiO_2_ film through the drop-casting method. Figure 2b represents the optical transmittance spectra of the RS layer without (w/o) or with (w/) the embedded SWCNT random network on the glass substrate. The insets depict the optical transmittance in the visible light wavelength spectrum (400–800 nm) for each sample and a photographic image of the sample w/ the embedded SWCNT random network. The optical transmittance for the RS layer w/ the embedded SWCNT random network increases at a lower wavelength value than the sample w/o the embedded SWCNT random network. In addition, the average optical transmittance at the visible-light region is 90.2% and 90.9% for the RS layers w/o and w/ the embedded SWCNT random network, respectively. Thus, the optical transmittance increases with the SWCNT random network embedment.

### 2.3. Characterization Methods

The RS operations and memristive synapse behaviors of the fabricated devices were analyzed using the Agilent HP 4156B precision semiconductor parameter analyzer (Hewlett-Packard Corp., Palo Alto, CA, USA), and electrical pulse stimulation was applied by the Agilent 8110A pulse generator (Hewlett-Packard Corp., USA). The devices were positioned on a two-point probe system in a dark box to minimize light and electrical noise. The surface topography of the SWCNT random network was analyzed by an AFM (SPM Solver Pro, NT-MDT Spectrum Instruments, Moscow, Russia). In addition, the optical transmittances for the RS layers were measured in the wavelength spectra region of 190–1100 nm with the Agilent 8453 ultraviolet-visible spectrophotometer (Hewlett-Packard Corp., USA).

## 3. Results and Discussion

### 3.1. RS Operations

**Figure 3:**
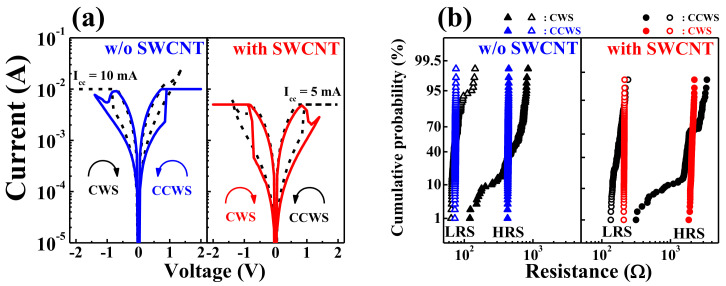
RS operations of HSQ memristors w/o and w/ the embedded SWCNT random network: (**a**) typical coexistence BRS operation *I*–*V* curves in clockwise switching (CWS) and counter-clockwise switching (CCWS) operations and (**b**) cumulative resistance probability during 10^2^ DC cycles in CWS and CCWS operations.

Figure 3a shows the current–voltage (*I*–*V*) curves of the fabricated HSQ memristor devices w/o and w/ the embedded SWCNT random network, all of which show typical bipolar RS (BRS) behavior. The measurement of the BRS *I*–*V* characteristics was performed by applying a sequential DC voltage to the TE with the BE grounded, as shown in Figure 1a. In general, BRS modes can be divided into clockwise switching (CWS) and counterclockwise switching (CCWS). In CWS, the set operation occurs at the negative bias, and the reset operation occurs at the positive bias. Conversely, in CCWS, the set and reset operations occur in the opposite polarities in CWS [37,38,39].

We measured the BRS *I*–*V* characteristics by applying a sequential DC bias voltage of 0 V → −2 V → 0 V → 1.4 V → 0 V for the CWS measurements and 0 V → 2 V → 0 V → −1.4 V → 0 V for the CCWS measurements. The HSQ memristors represented a typical coexistence BRS operations with CWS and CCWS behaviors in one cell, as shown in Figure 3a. Meanwhile, a stable BRS was obtained at a set compliance current (*I_cc_*) of 10 × 10^−3^ A w/o the embedded SWCNT random network but at an *I_cc_* of 5 × 10^−3^ A w/ the embedded SWCNT random network. Figure 3b shows the resistance uniformity over 10^2^ DC cycles in CWS and CCWS modes for HSQ memristors w/o and w/ the embedded SWCNT random network. We compared the cumulative probabilities of resistance to determine the correlation between the two memristors and DC voltage sweep modes. Interestingly, the stability of the switching characteristics was affected by the direction of the voltage sweep depending on the presence or absence of the SWCNT random network. The resistance values of the high-resistance state (HRS) and low-resistance state (LRS) were extracted from the read voltages of −0.2 V (in CWS) and 0.2 V (in CCWS), respectively, for each cycle. Table 1 and Table 2 summarize the statistical resistance values in five HSQ memristors w/o and w/ the embedded SWCNT random network which operated in CWS and CCWS mode, respectively. As a result, the memristor device w/o the embedded SWCNT random network appeared more stable in the CCWS mode, whereas w/ the embedded SWCNT random network was more stable in the CWS mode.

**Figure 4:**
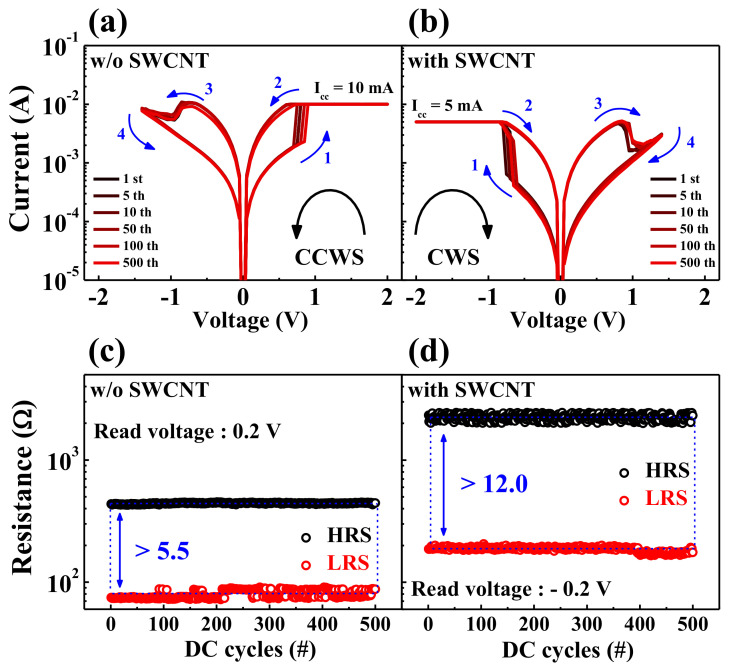
RS endurance characteristics of HSQ memristors w/o and w/ the embedded SWCNT random network for 5 × 10^2^ DC cycles. BRS *I*–*V* curves of the HSQ memristors (**a**) w/o and (**b**) w/ the embedded SWCNT random network. Resistance endurance of the HSQ memristors (**c**) w/o and (**d**) w/ the embedded SWCNT random network.

Figure 4a,b show the results of a 5 × 10^2^ DC cycle endurance test on the HSQ memristors for a reliability evaluation, where the HSQ memristors w/o and w/ the embedded SWCNT random network are operated by CCWS and CWS, respectively. Figure 4c,d show the resistance values of HRS/LRS (*R_HRS_*/*R_LRS_*) during the endurance test determined at read voltages of 0.2 V (for the device w/o the SWCNT random network) and −0.2 V (for the device w/ the SWCNT random network), respectively. The average *R_HRS_*/*R_LRS_* and standard deviation (SD) of the HSQ memristor w/o the embedded SWCNT random network were 441.7 Ω (SD = 4.5 Ω)/79.2 Ω (SD = 5.8 Ω). Conversely, the average *R_HRS_*/*R_LRS_* and SD in HSQ memristor w/ the embedded SWCNT random network were 2239.6 Ω (SD = 120.1 Ω)/186.4 Ω (SD = 7.6 Ω). These results indicate that both HSQ memristors have stable BRS endurance over 5 × 10^2^ DC cycles, and the RS windows, which can be defined as the resistance ratio between *R_HRS_* and *R_LRS_*, are enlarged from >5.5 to >12.0 due to the embedded SWCNT random network. Because SWCNTs have highly responsive structural defects in the hexagonal rings of their carbon atoms, the SWCNT random network triggers more oxygen adsorption and desorption. Thus, the embedded SWCNT random network layer can tune the interface dynamics and produce oxygen vacancy-rich conductive filaments in the HSQ thin film [40,41,42,43].

**Figure 5:**
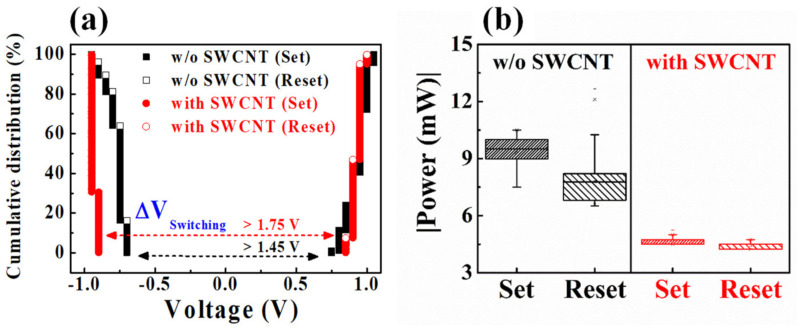
(**a**) Cumulative distribution of the operating voltage and (**b**) operating powers of HSQ memristors w/o and w/ the embedded SWCNT random network.

Figure 5a shows the cumulative set voltage (*V_set_*) and reset voltage (*V_reset_*) distribution of HSQ memristor devices w/o and w/ the embedded SWCNT random network. *V_set_* can be regarded as the voltage at the point where the conductance of the BRS *I*–*V* curve abruptly changes from HRS to LRS. Conversely, *V_reset_* can be defined from the reset current (*I_reset_*), which is the peak current point when the conductance in the BRS *I*–*V* curve begins to decrease during the reset operation [44]. The HSQ memristor w/o the embedded SWCNT random network has an average *V_set_*, *V_reset_*, and SD values of *V_set_* = 0.92 V (SD = 0.07 V) and *V_reset_* = −0.77 V (SD = 0.06 V). Meanwhile, the HSQ memristor w/ the embedded SWCNT random network has *V_set_* = –0.93 V (SD = 0.02 V) and *V_reset_* = 0.92 V (SD = 0.04 V). Figure 5b represents the power for the set (*P_set_*) and reset (*P_reset_*) operations of the HSQ memristor devices w/o and w/ the embedded SWCNT random network, where *P_set_* and *P_reset_* are defined as *P_set_* = *V_set_* × *I_cc_* and *P_reset_* = *V_reset_* × *I_reset_*, respectively. The average *P_set_* and *P_reset_* of the HSQ memristor w/o the embedded SWCNT random networks were 9.27 and 7.75 mW, respectively. Conversely, for the HSQ memristor w/ the embedded SWCNT random network, the average *P_set_* and *P_reset_* are 4.67 and 4.46 mW, respectively. Table 3 summarizes the electrical parameters of the RS operating characteristics for the presented HSQ memristors w/o and w/ the embedded SWCNT random network.

As shown in Figure 4 and Figure 5, the HSQ memristor w/ the embedded SWCNT random network has a larger RS window at a lower *I_cc_* of 5 × 10^−3^ A, more uniform *V*_set_–*V*_reset_ distribution, and lower operating power than the HSQ memristor w/o the embedded SWCNT random network. In addition, there is a sufficient voltage margin (>1.75 V) between *V_set_* and *V_reset_* (Δ*V_Switching_* = minimum *V_set_* − maximum *V_reset_*). Therefore, the SWCNT random network embedded in the HSQ thin film significantly contributed to the RS performance improvement.

### 3.2. Current Transport Mechanism

**Figure 6:**
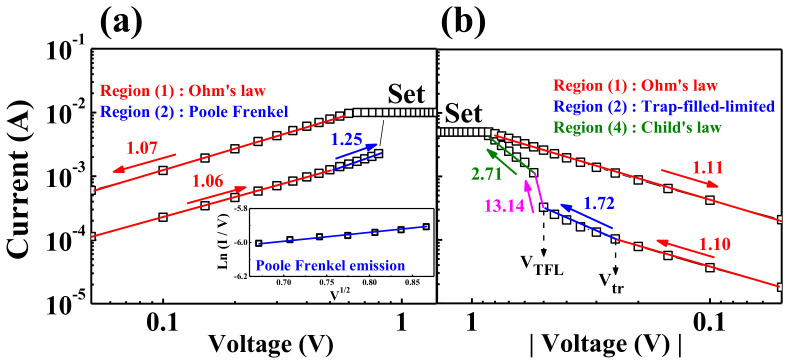
Current transport mechanism of the double logarithmic plot *I*–*V* curves in the set operation. (**a**) Poole–Frenkel conduction mechanism of the HSQ memristor w/o the embedded SWCNT random network. Inset: fitting by the *ln(I/V)–V^1/2^* relationship. (**b**) Space-charge-limited conduction mechanism of the HSQ memristor w/ the embedded SWCNT random network.

To investigate the improved RS characteristics due to the embedded SWCNT random network, the current transport mechanisms were analyzed by plotting the BRS *I*–*V* curves of the set operation region on a log–log scale. Figure 6 shows the double logarithmic plot of the *I*–*V* curves for the HSQ memristors w/o or w/ the embedded SWCNT random network.

Particularly, in the HRS, the HSQ memristor w/o the SWCNT random network in Figure 6a shows two different conduction mechanisms depending on the applied voltage. In Figure 6a, the HSQ memristor w/o the SWCNT random network follows the Ohmic conduction mechanism in the low-voltage regime of the HRS. The slope of the *I*–*V* curve is close to “1” because the trap energy level in the RS layer prevents electrons from overcoming the high trap energy. However, as the applied voltage in the HRS increases, the current increases nonlinearly through the lower trap barrier [45]. As shown in the inset of Figure 6a, the device w/o the SWCNT random network follows the *ln*(*I*/*V*)–*V*^1/2^ relationship given by Equation (1), which implies the Poole–Frenkel conduction mechanism in the high-voltage regime of the HRS:

(1) $$J\propto qEexp\left[\frac{-q\left(\emptyset_{T}-\sqrt{qE/\pi \varepsilon }\right)}{kT}\right],$$

where *J* is the current density, *q* is the electronic charge, *E* is the electric field, *q∅_T_* is the trap energy level, *ε* is the permittivity, *K* is the Boltzmann constant, and *T* is the absolute temperature [46]. After the set operation, the resistance state of the RS layer is switched to the LRS through the conductive filament connection, and the Ohmic characteristic of the LRS is maintained until the reset operation.

Conversely, in the case of the HSQ memristor w/ the SWCNT random network in Figure 6b, the *I*–*V* curve for the HRS shows a different behavior from Figure 6a. In the low-voltage regime of the HRS, indicated by regime (1), this device also follows the Ohmic conduction mechanism. In the low-voltage regime of the HRS shown in regime (1), the device follows an Ohmic conduction mechanism, where the thermally generated free charge density prevails over a small number of injected carriers in the RS layer because the electric field applied to the RS layer is insufficient. However, as the voltage increases, the current increases nonlinearly, and there are three additional regimes in which the slope of the curve changes: trap-filled-limited (TFL) behavior in regime (2), rapid current increase in regime (3), and Child’s law in regime (4) [46]. In regime (2), the Ohmic-like behavior transforms to the TFL behavior at the transition voltage (*V_tr_*). As the applied voltage increases, the injected carrier density exceeds the thermally generated carriers, and then the current increases nonlinearly. However, the shallow traps in the RS layer are occupied by the injected carrier, and the current flow is limited by the *I–V^2^* relationship. When the shallow traps are filled with carriers, the slope of the current sharply increases, which corresponds to the TFL voltage (*V_TFL_*). In regime (4), space charges are formed by trapped carriers and current flows in the *I–V^2^* relationship according to Child’s law [46,47,48]. The current density of each regime can be represented by Equations (2)–(4):

(2) $$J_{Ohm}=qn_{0}\mu \frac{V}{d}.$$

(3) $$J_{TFL}=\frac{9}{8}\mu \varepsilon \theta \frac{V^{2}}{d^{3}}.$$

(4) $$J_{Child}=\frac{9}{8}\mu \varepsilon \frac{V^{2}}{d^{3}}.$$

### 3.3. Multilevel RS Operations

**Figure 7:**
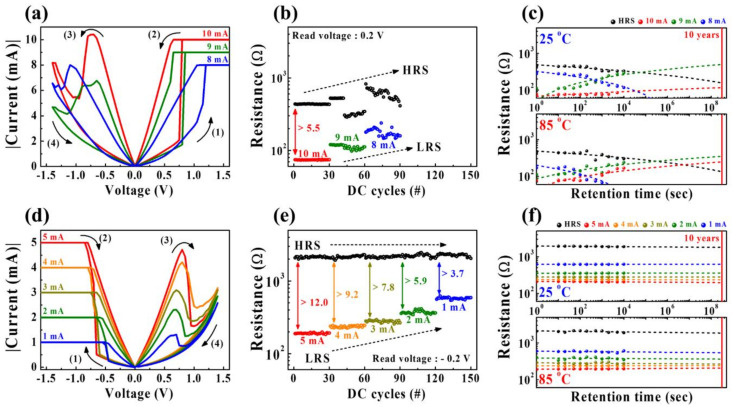
Multilevel RS characteristics through *I_cc_* modulation for HSQ memristors w/o (**a**–**c**) and w/ (**d**–**f**) the embedded SWCNT random network. (**a,d**) Multilevel BRS *I*–*V* curves. (**b,e**) Multiple-resistance state endurance characteristics. (**c,f**) Nonvolatile multiple-resistance state retention characteristics for 10^4^ s at room temperature (25 °C) and high temperature (85 °C).

Figure 7 shows the multilevel RS characteristics for the HSQ memristors w/o or w/ the embedded SWCNT random network. Memristor devices have received attention for memory applications, but recently they have also been studied as artificial synapses for neuromorphic applications. Therefore, nonvolatile multiple-resistance states in a single memristor cell are essential for analog synaptic weight modulation, which can be achieved by gradually increasing and decreasing conductance by applying proper electrical signals. For conductive filament-based memristors, multiple-resistance states can be modulated through the conductive filament widening in the RS layer by adjusting the *I*–*V* measurement conditions [49,50].

Figure 7a,d show the multilevel BRS characteristics via *I_cc_* modulation for HSQ memristors w/o and w/ the embedded SWCNT random network, respectively. In Figure 7a, w/o the embedded SWCNT random network, the HSQ memristor exhibits an unstable RS behavior as *I_cc_* changes (range from 8 to 10 mA). Conversely, w/ the embedded SWCNT random network in Figure 7d, the HSQ memristor exhibits several stable LRS levels depending on the modulation of *I_cc_* (ranging from 1 to 5 mA). Figure 7b,e show the endurance characteristics of multiple-resistance states recorded through 30 DC cycle tests under the corresponding *I_cc_* conditions for HSQ memristors w/o and w/ the embedded SWCNT random network, respectively. For HSQ memristors w/o the embedded SWCNT random networks in Figure 7b, unstable multiple-resistance state endurance was observed. The levels of the LRS and HRS were not constant with increasing DC cycles, especially under low *I_cc_* conditions. Conversely, in the case of the HSQ memristor w/ the embedded SWCNT random network, shown in Figure 7e, the levels of the LRS and HRS remained almost constant with increasing DC cycles, providing excellent endurance characteristics. Figure 7c,f show the retention characteristics for multiple-resistance states of the HSQ memristors w/o and w/ the embedded SWCNT random network, respectively, measured at room temperature (25 °C) and high temperature (85 °C) for 10^4^ s. For the HSQ memristors w/o the embedded SWCNT random network, shown in Figure 7c, the multiple-resistance states are all rapidly degraded, showing a lack of nonvolatility. Meanwhile, in Figure 7e, for the HSQ memristor w/ the embedded SWCNT random network, the multiple-resistance states remained unchanged for 10^4^ s at the both measured temperatures, providing sufficient nonvolatility. Therefore, we verified that the multilevel RS operation of the HSQ memristor device was significantly improved by the embedded SWCNT random network. Particularly, this reliable multilevel resistance modulation characteristic indicates the possibility of mimicking biological synapses.

### 3.4. Synaptic Weight Modulation

**Figure 8:**
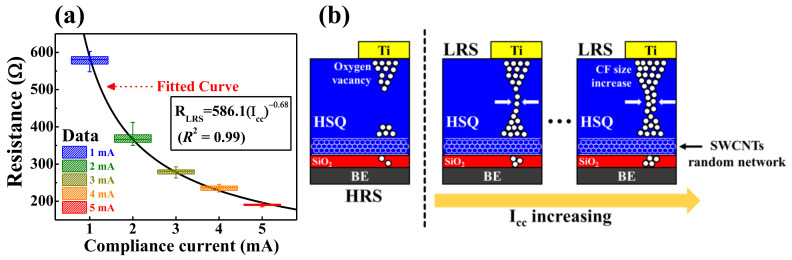
HSQ memristor w/ the embedded SWCNT random network: (**a**) Dependence of the average *R_LRS_* values according to the magnitude of *I_cc_* and (**b**) simple schematics of the conductive filament formation in the RS layer of the SWCNT random network embedded in the HSQ thin-film nanocomposite.

Figure 8a shows the dependence of the average *R_LRS_* values according to the magnitude of *I_cc_*, and Figure 8b presents simple schematic diagrams of the formation of conductive filaments in the RS layer of an HSQ memristor w/ the embedded SWCNT random network. Because the conductance modulation of a conductive filament-based memristor device has considerable analog synaptic weight variability between adjacent synaptic connections, conductive filament-based memristors are considered suitable candidates for electronic synaptic systems [51,52,53,54]. This study verified that the HSQ memristor w/ the embedded SWCNT random network has stable multilevel resistance modulation characteristics, and the average *R_LRS_* value is stably modulated according to the magnitude of *I_cc_*. In addition, the average *R_LRS_* decreases exponentially with an increase in *I_cc_*, which was fitted in the relationship of *R_LRS_* ∝ (*I_cc_*)^−0.68^, as indicated by a solid line in Figure 8a. As a result, a well-fitted *R_LRS_* relation indicates the possibility of an analog synaptic weight modulation [55,56]. Figure 8b provides schematic diagrams for the conductive filament formation process in the RS layer of the SWCNT random network embedded in the HSQ thin-film nanocomposite. The embedded SWCNT random network triggers the oxygen vacancy-rich conductive filament in the LRS state, and the size of the conductive filament increases with increasing *I_cc_*. Since the SWCNT random network has highly responsive structural defects in their carbon atoms, the SWCNTs can trigger more oxygen adsorption and desorption [40,42]. When applied the negative bias to the TE, the negatively charged oxygen ions can drift to the SWCNTs and adsorbed, resulting in the oxygen vacancy and an SWCNTs type transition into the p-type property. Conversely, by applying the positive bias to the TE, the desorbed oxygen ions diffuse back to the conductive filament in the RS layer and neutralize the oxygen vacancy. Therefore, CWS operation in the HSQ memristor w/ the embedded SWCNT random network has stable RS operations than CCWS mode due to the interfacial dynamics by SWCNT embedment [37,38,39,40].

**Figure 9:**
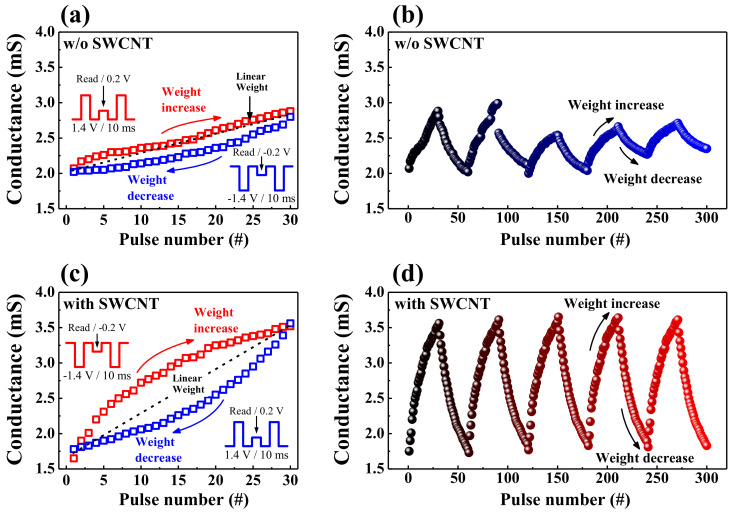
Synaptic weight potentiation/depression for HSQ memristors w/o (**a**,**b**) and w/ (**c**,**d**) the embedded SWCNT random network: (**a**,**c**) one-cycle properties of consecutive conductance modulation (insets depict the synaptic pulse scheme of potentiation, depression, and read operations) and (**b**,**d**) weight modulation reliability for 3 × 10^2^ synaptic pulses.

Figure 9 shows the analog synaptic weight modulation properties to evaluate the continuous RS operation in the HSQ memristor w/o or w/ the embedded SWCNT random network. The potentiation (increasing conductance) and depression (decreasing conductance) processes of the presented devices can be emulated by an easily implementable algorithm based on a train of identical pulses (synapse stimulation spikes). Figure 9a,c exhibit the one-cycle (30 pulses each for potentiation and depression) of consecutive conductance modulation for HSQ memristors w/o and w/ the embedded SWCNT random network, respectively. The synaptic pulse conditions for potentiation and depression were 1.4 and −1.4 V/10 ms, respectively. For the HSQ memristor w/o the SWCNT random network, the dynamic range of conductance variation was ~0.7 mS, and the asymmetric weight increase/decrease behavior was observed, as shown in Figure 9a. Furthermore, the memristor device w/ the SWCNT random network has a wider range of conductance variation of ~2.0 mS and revealed a more symmetric weight modulation behavior than the memristor w/o the SWCNT random network, as shown in Figure 9c. Figure 9b,d show the consecutive weight increase and decrease characteristics for 3 × 10^2^ synaptic pulses to evaluate the reliability of the weight modulation. As shown in Figure 9b, the conductance modulation of the HSQ memristor w/o the SWCNT random network was unstable, and the dynamic range was reduced to ~0.5 mS. Conversely, the conductance of the HSQ memristor w/ the SWCNT random network was reliably modulated over 3 × 10^2^ synaptic pulses without deteriorating the dynamic range, as shown in Figure 9d. Consequently, we verified that the embedded SWCNT random network stabilized the synaptic weight modulation characteristics and contributed to improving the reliability of HSQ memristor devices.

## 4. Conclusions

In this paper, we propose HSQ memristors embedded with an SWCNT random network as suitable devices for flexible artificial synapses in future neural form circuits. Flexible memristors were fabricated on a PEN substrate using a low-temperature solution process compatible with SWCNT random networks embedded in HSQ nanocomposites as the RS layer. Then, we systematically investigated the effects of the embedded SWCNT random network on the RS operation and synaptic behavior of HSQ-based memristor devices. The high gap-fill ability of the HSQ allowed the formation of an RS layer embedded with a homogeneous SWCNT random network. The embedded SWCNT random network triggered the oxygen vacancy-rich conductive filament in the RS layer. The fabricated HSQ-based memristors exhibited the coexistence of the BRS operation, representing the CWS and CCWS operations. However, the embedded SWCNT random network reversed the stable RS operating polarity: the HSQ memristor w/o the SWCNT random network was more stable in the CCWS operation, whereas the HSQ memristor w/ the SWCNT random network was more stable in the CWS operation. In addition, the embedded SWCNT random network featured a larger RS window, a more uniform *V*_set_ − *V*_reset_ distribution, and lower operating power. We analyzed the different RS polarities according to the presence of the SWCNT random network by the current transport mechanism. Furthermore, the embedded SWCNT random network facilitated stable multilevel RS performances. Multiple-resistance states exhibited stable endurances and highly reliable nonvolatile retention over 10^4^ s even at a high temperature (85 °C), suggesting the possibility of an analog synaptic weight modulation. Consequently, the synaptic weights were gradually modulated through 3 × 10^2^ stimulation pulses, demonstrating the high effect of the embedded SWCNT random network on the RS layer of the memristor. Therefore, HSQ nanocomposites with SWCNT random networks are expected to be useful for emerging flexible artificial electronic synapses owing to their material versatility and low-temperature solution processability.

## Figures and Tables

**Table 1:** Summary of the statistical resistance values in five HSQ memristors w/o the embedded SWCNT random network.

w/o SWCNTs (CWS Mode)						
Device number		1	2	3	4	5
Resistance (HRS)	Mean value (*μ*)	541.2 Ω	541.4 Ω	541.7 Ω	540.6 Ω	540.9 Ω
	Standard deviation (*σ*)	197.2 Ω	196.8 Ω	369.8 Ω	185.8 Ω	239.4 Ω
	Statistical value	541.1 ± 237.8 Ω				
Resistance (LRS)	Mean value (*μ*)	79.9 Ω	80.0 Ω	79.8 Ω	79.8 Ω	80.0 Ω
	Standard deviation (*σ*)	19.2 Ω	19.5 Ω	22.0 Ω	20.7 Ω	16.5 Ω
	**Statistical value**	**79.9 ± 19.5 Ω**				
**w/o SWCNTs (CCWS Mode)**						
Device number		1	2	3	4	5
Resistance (HRS)	Mean value (*μ*)	440.7 Ω	440.6 Ω	440.9 Ω	440.4 Ω	440.6 Ω
	Standard deviation (*σ*)	3.8 Ω	3.0 Ω	4.6 Ω	3.4 Ω	6.2 Ω
	**Statistical value**	**441.2 ± 4.2 Ω**				
Resistance (LRS)	Mean value (*μ*)	74.3 Ω	74.3 Ω	74.4 Ω	74.3 Ω	74.2 Ω
	Standard deviation (*σ*)	0.6 Ω	1.8 Ω	4.2 Ω	5.9 Ω	5.3 Ω
	**Statistical value**	**74.3 ± 3.5 Ω**				

**Table 2:** Summary of the statistical resistance values in five HSQ memristors w/ the embedded SWCNT random network.

w/ SWCNTs (CWS Mode)						
Device number		1	2	3	4	5
Resistance (HRS)	Mean value (*μ*)	2160.8 Ω	2112.6 Ω	2165.5 Ω	2167.2 Ω	2179.1 Ω
	Standard deviation (*σ*)	80.9 Ω	103.8 Ω	71.9 Ω	100.3 Ω	71.6 Ω
	Statistical value	2157.0 ± 85.7 Ω				
Resistance (LRS)	Mean value (*μ*)	192.4 Ω	192.7 Ω	192.5 Ω	193.6 Ω	195.8 Ω
	Standard deviation (*σ*)	1.3 Ω	4.7 Ω	21.4 Ω	2.6 Ω	9.1 Ω
	**Statistical value**	**193.4 ± 7.8 Ω**				
**w/ SWCNTs (CCWS Mode)**						
Device number		1	2	3	4	5
Resistance (HRS)	Mean value (*μ*)	1867.5 Ω	1870.1 Ω	1868.8 Ω	1870.6 Ω	1879.2 Ω
	Standard deviation (*σ*)	716.0 Ω	495.7 Ω	873.0 Ω	636.0 Ω	877.9 Ω
	**Statistical value**	**1871.2 ± 719.7 Ω**				
Resistance (LRS)	Mean value (*μ*)	174.5 Ω	175.1 Ω	175.5 Ω	174.9 Ω	175.5 Ω
	Standard deviation (*σ*)	24.8 Ω	7.6 Ω	31.0 Ω	38.5 Ω	31.0 Ω
	**Statistical value**	**175.1 ± 26.5 Ω**				

**Table 3:** Electrical parameters for the RS operating characteristics of the HSQ memristors w/o and w/ the embedded SWCNT random network.

Switching Layer	Stable RS Polarity	Endurance			Distribution		Power	
		*R_HRS_* [Ω] (SD)	*R_LRS_* [Ω] (SD)	RS Window	*V_set_* [V] (SD)	*V_reset_* [V] (SD)	*P_set_* [mW]	*P_reset_* [mW]
w/o SWCNTs	CCWS	441.7 (4.5)	79.2 (5.8)	>5.5	0.92 (0.07)	−0.77 (0.06)	9.27	7.75
w/ SWCNTs	CWS	2239.6 (120.1)	186.4 (7.6)	>12.0	−0.93 (0.02)	0.92 (0.04)	4.67	4.46

## Data Availability

Not applicable.
